# Supplementary material for: Exploring the Interplay between Metabolic Pathways and Taxane Production in Elicited Taxus baccata Cell Suspensions
Source: Plants (Basel). 2023 Jul 19;12(14):2696. doi: 10.3390/plants12142696 (PMC10386569; doi:10.3390/plants12142696)
Supplement: Supplementary file 1 [file plants-12-02696-s001.zip › plants-2505120-supplementary.pdf]

Supplementary Material

Exploring the interplay between metabolic pathways and taxane production from elicited *Taxus baccata* cell suspensions.

Edgar Perez-Matas[1], Pascual Garcia-Perez[2,3], Begoña Miras-Moreno[2], Luigi Lucini[2], Mercedes Bonfill[1], Javier Palazon[1*] and Diego Hidalgo-Martinez[1*]

[1] Department of Biology, Healthcare and the Environment, Faculty of Pharmacy and Food Sciences, University of Barcelona, 08028 Barcelona, Spain. epm093@hotmail.com (E.P.-M.); mbonfill@ub.edu (M.B.)

[2] Department for Sustainable Food Process, Università Cattolica Del Sacro Cuore, Via Emilia Parmense 84, 29122, Piacenza, Italy. pasgarcia@uvigo.es (P.G.-P.); mariabegona.mirasmoreno@unicatt.it (B.M-M); luigi.lucini@unicatt.it (L.L.).

[3] Nutrition and Bromatology Group, Department of Analytical and Food Chemistry, Faculty of Food Science and Technology, Ourense Campus, Universidade de Vigo,E32004 Ourense, Spain

* Correspondence: dhidalgo@ub.edu (D.H.-M.); javierpalazon@ub.edu (J.P.).

Table S1. Sequences of the primers used to amplify the genes by RT-qPCR.

Gene	*Primer sequences*	*Amplicon size*	*Reference*
GGPPS	Forward: 5'-CCG GTG TGT GGG GCT TCT GTT T-3'<br>Reverse: 5'-TTT GCT TTC TCC AGG CCC ATC A-3'	144 bp	[1]
TXS	Forward: 5'-TTC GCA CGC ACG GAT ACG-3'<br>Reverse: 5'-TTC ACC ACG CTT CTC AAT TCG-3'	115 bp	[2]
BAPT	Forward: 5'-TAA GCA CTC TAC AAC AAC AGG-3'<br>Reverse: 5'-GCA TGA ACA TTA GTA TCT TGA TTC C-3'	111 bp	[2]
T7βOH	Forward: 5'-GGT CCG CCC AAA TTG CCA GAA-3'<br>Reverse: 5'-CCC TGC AGA GCC CAA AAA ACC-3'	110 bp	[2]
DBAT	Forward: 5'-AGT TGG ATT TGG TGA TCG AA-3'<br>Reverse: 5'-ATC CAT GTT GCA CGA GAC TT-3'	92 bp	[1]
DBTNBT	Forward: 5'-CGG GGG GTT TGT TGT GGG ATT A-3'<br>Reverse: 5'-TTA GCC TCT CCC CTC GCC ATC T-3'	105 bp	[1]
PAM	Forward: 5'-CCC GGA GGC ATG ACG TGA AG-3'<br>Reverse: 5'-CGC CGT CTT CCG CCT TGC-3'	99 bp	[1]
TBC41	Forward: 5'-CAA GAA GAA AGA GTC AGC AAA TGG-3'<br>Reverse: 5'-GGA ACG ACA TGA CAT TAT GAA TAG C-3'	91 bp	[3]

Table S2. Selected metabolites after statistical and FC analyses (day 8).

Category	Metabolite	Log FC ([COR] vs [Control])	Log FC ([SA] vs [Control])
Nitrogen-Containing Secondary Compound Biosynthesis	N-feruloyltyramine	-10.13	-10.13
	5-hydroxy-γ-coniceine	1.22	-1.1
	coniferyl alcohol	-2.81	0.31
	(S)-6-O-methylnorlaudanosoline	-10.13	0.24
	piperine	-10.13	-1.06
	caffeoylserotonin	10.13	
	feruloylserotonin	3.77	-10.13
	camptothecin	1.86	0.62
	norbelladine	-3.19	-0.71
	4'-O-methylnorbelladine	-10.13	-0.91
	chelerythrine	1.84	0.63
	agmatine	-10.13	-1.69
	4-coumaroylagmatine	-10.13	-3.44
	7'-O-demethylcephaeline	-10.13	2.91
	protoemetine	-2.74	-1.51
	6,7-O,O-dimethyl-N-deacetylisoipecoside aglycon		10.13
	6-O-methyl-N-deacetylisoipecoside aglycon		10.13
	vindoline	-10.13	0.38
	vindorosine	-10.13	-1.26
	(Z)-1-(glutathion-S-yl)-N-hydroxy-2-phenylethan-1-imine	10.13	
	(Z)-1-(L-cysteinyglycin-S-yl)-N-hydroxy-2-phenylethan-1-imine	-10.13	-0.55
	3-(aminomethyl)indole	1.65	0.43
	N-formyldemecolcine	6.03	2.53
	N-hydroxyhomomethionine	1.22	0.31
	L-homomethionine	10.13	
	(E)-4-(methylsulfanyl)butanal oxime	-10.13	-0.44
	(E)-1-(glutathion-S-yl)-N-hydroxy-ω-(methylsulfanyl)butan-1-imine	6.04	2.87
	dehydroscoulerine	4.05	0.65
	ajmaline	-10.13	0.78
	17-O-acetylajmaline		10.13
	16-epivellosimine	-4.98	-3.95
	17-O-acetylnorajmaline		10.13
	guattegaumerine	1.25	-0.21
	scopolamine	6.76	0.43
	(6S)-hydroxyhyoscyamine	-3.5	-0.84
	N-hydroxypentahomomethionine	-10.13	0.35
	N,N-dihydroxypentahomomethionine	-10.13	0.6
	N, N-dihydroxy-tetrahomomethionine	3.07	1.53
	(E)-7-(methyltsulfanyl)heptanal oxime	10.13	
	N-hydroxy-L-phenylalanine	1.18	0.11
	N,N-dihydroxy-L-phenylalanine	-1.4	0.02
	(R)-prunasin	-10.13	-0.8
	4-(1-methyl-2-pyrrolidinyl)-3-oxobutanoate methyl ester	3.81	1.23
	quinidinone	-10.13	-1.17
	quinidine	4.82	4.6
	(S)-cis-N-methylstylopine	-1.53	-0.64
	chelirubine	-10.13	-0.68
	2-[(5'-methylsulfanyl)pentyl]malate	-10.13	-10.13
	3-carboxy-10-(methylsulfanyl)-2-oxodecanoate	-10.13	-0.33
	L-hexahomomethionine	-10.13	-1.28
	6-(methylsulfanyl)-2-oxohexanoate	10.13	10.13
	(S)-stylopine	10.13	
	canavaninosuccinate	-10.13	-0.66
	L-canaline	10.13	10.13
	lotaustralin	-1.87	-0.6
	7-deoxyloganetate	10.13	
	(6E)-8-hydroxygeraniol	-10.13	2.44
	trigonelline	10.13	10.13
	(R)-3,4-dihydroxymandelonitrile	-10.13	-0.92
	dopaquinone	10.13	10.13
	1-O-feruloyl-β-D-glucose	2.06	0.79
	betanidin	10.13	
	miraxanthin V	-10.13	-1.19
	betalamate	1.37	-0.48
	(indol-3-yl)acetaldehyde	10.13	10.13
	1,2-dihydropyrimidine	10.13	10.13
	3,6-dihydronicotine	10.13	
	7,8-dihydroberberine	4.73	1.41

continuation of Table S2

Category	Metabolite	Log FC ([COR] vs [Control])	Log FC ([SA] vs [Control])
Nitrogen-Containing Secondary Compound Biosynthesis	(S)-magnoflorine	-10.13	-0.92
	(S)-corytuberine	-3.5	-0.84
	dhurrin	-10.13	-0.55
	13-hydroxylupanine	3.02	0.43
	(E)-6-(methylsulfanyl)hexanal oxime	1.09	-0.1
	glutathione	2.12	0.31
	(E)-1-(L-cystein-S-yl)-N-hydroxy-ω-(methylsulfanyl)hexan-1-imine	4.73	1.89
	(E)-1-(L-cysteinylglycin-S-yl)-N-hydroxy-ω-(methylsulfanyl)hexan-1-imine	2.64	1.42
	O-acetyl-L-serine	1.21	0.32
	(R)-laudanine	-4.18	1.04
	tyramine	3.84	1.66
	L-tyrosine	-10.13	0.34
	(S)-canadine	-1.93	-0.73
	(S)-tetrahydrocolumbamine	-4.58	-0.65
	(13S,14R)-13-O-acetyl-1-hydroxy-N-methylcanadine	-10.13	-10.13
Phenylpropanoid Derivative Biosynthesis	coniferyl acetate	0.86	-2.31
	6-gingerol	-10.13	-1.58
	1-(3,4-dihydroxyphenyl)-5-hydroxy-3-decanone	-4.61	-0.64
	afrormosin-7-O-glucoside	10.13	
	1-O-caffeoyl-β-D-glucose	10.13	
	(-)-gallocatechin-3-O-gallate	10.13	10.13
	(+)-sesaminol	1.82	0.62
	dihydropinosylvin	-1.89	-0.41
	(-)-phaseollin	1.52	-0.44
	syringin	10.13	10.13
	dihydroconiferyl alcohol glucoside	1.93	0.97
	trans-coutarate	-10.13	-1.72
	(S)-dihydrodaidzein	1.97	0.91
	benzoyl-β-D-glucopyranose	2.12	0.31
	5-hydroxy-coniferaldehyde	3.59	1.6
	(6αS,11αS)-4-dimethylallyl-3,6α,9-trihydroxypterocarpan	2.08	0.81
	O-sinapoylcholine	-10.13	-0.64
	(3R,4R)-7,2',4'-trihydroxyisoflavanol	-10.13	-0.33
	(3R,4R)-3,4-bis(4-hydroxy-3-methoxybenzyl)tetrahydro-2-furanol	-10.13	-10.13
	eugenol O-β-D-xylopyranosyl-(1->6)-O-β-D-glucopyranoside	-10.13	-10.13
	1,4-dihydroxy-2-naphthoate	-10.13	0.4
	(-)-thujaplicatin	3.71	1.65
	O4,O5--dimethylthujaplicatin	2.07	0.35
	eugenol	1.73	0.49
	4-O-β-D-glucosyl-esculetin	2.06	0.2
	3,7,3',4'-tetramethylquercetin	2.96	1.58
	daphnetin	2.3	-0.04
	apigenin 7-O-β-D-glucoside	1.01	0.29
	(2R,3S,4S)-leucodelphinidin		10.13
	luteone	2.64	1.45
	1-O-feruloyl-β-D-glucose	2.06	0.79
	diphyllin	2.96	1.58
	3-geranyl-4-hydroxybenzoate	-10.13	-0.91
	3''-hydroxy-geranylhydroquinone	-10.13	-0.76
	deoxyshikonin	10.13	
	p-coumaroyltriacetate	10.13	10.13
	rot-2'-enonate		10.13
	(3R,4R)-7,2'-dihydroxy-4'-methoxyisoflavanol	5.43	3.08
	2'-O-methylisoliquiritigenin	10.13	
	coniferyl alcohol	-2.81	0.31
	coniferin	2.96	1.58
	trans-5-O-caffeoyl-D-quinate	2.64	1.42
	t-anethole	2.08	0.6
	5'-demethoxy-6-methoxypodophyllotoxin	10.13	
	6-methoxypodophyllotoxin	10.13	
	6-methoxypodophyllotoxin 7-glucoside	-10.13	-0.4
	sorgoleone		10.13
	4-coumaryl alcohol	10.13	
	(7S)-cis-hinokiresinol	-1.5	-0.12
	4-coumaryl-4-coumarate	10.13	
	L-tyrosine	-10.13	0.34
	malonate	2.05	0.22
	quercetin 3-O-(6''-O-p-coumaroyl)-glucoside	1.37	0.43
	3,6,7,3',4'-pentamethylquercetagetin	10.13	10.13
	3,6,7-trimethylquercetagetin	-10.13	-1.19

continuation of Table S2

Category	Metabolite	Log FC ([COR] vs [Control])	Log FC ([SA] vs [Control])
Phytoalexin Biosynthesis	(6αS,11αS)-4-dimethylallyl-3,6α,9-trihydroxypterocarpan	2.08	0.81
	3-hydroxy-5-methoxybiphenyl	10.13	10.13
	noraucuparin	10.13	
	p-coumaroyltriacetate	10.13	10.13
	luteone	2.64	1.45
	(3R,4R)-7,2'-dihydroxy-4'-methoxyisoflavanol	5.43	3.08
	ent-3β-hydroxy-cassa-12,15-diene-11-one	1.18	0.11
	geranylgeranyl diphosphate (GGPP)	-3.69	0.53
	kauralexin A2	5.67	6.54
	kauralexin A1	3.78	1.65
	15-hydroxysolavetivone	-2.35	-1.09
	3-hydroxy-15-dihydrolubimin	-1.7	-0.55
	oryzalide A	-10.13	0.83
	(4S)-4-(5,5-dimethylcyclohex-1-en-1-yl)cyclohex-1-ene-1-carbaldehyde	-10.13	-0.46
	glutathione	2.12	0.31
	(L-cysteinylglycin-S-yl)(1H-indol-3-yl)acetonitrile	-10.13	-1.26
	(-)-phaseollin	1.52	-0.44
	(3R,4R)-7,2',4'-trihydroxyisoflavanol	-10.13	-0.33
	malonate	2.05	0.22
	2'-O-methylisoliquiritigenin	10.13	
	dihydropinosylvin	-1.89	-0.41
Sulfur-Containing Secondary Compound Biosynthesis	O-acetyl-L-serine	1.21	0.32
	petiveriin	-10.13	-1.17
	(Z)-butanethial S-oxide	0.35	-10.13
	thiosulfinate	-10.13	-2.29
	glutathione	2.12	0.31
Terpenoid Biosynthesis	10β,14β-dihydroxytaxa-4(20),11-dien-5α-yl acetate	1.13	0.05
	phytyl monophosphate	-10.13	-0.52
	sulcatone	10.13	
	bixin aldehyde		10.13
	norbixin	10.13	
	3β-hydroxyparthenolide	-10.13	0.71
	19-O-β-glucopyranosyl-steviol	-4.43	-0.37
	taxa-4,11-diene	-10.13	-10.13
	vindoline	-10.13	0.38
	vindorosine	-10.13	-1.26
	4α-carboxy-ergosta-7,24(241)-dien-3β-ol	-2.13	-1.17
	heliocide H1	-4.06	0.54
	heliocide B1	-10.13	0.2
	hemigossypolone-6-methyl ether	10.13	
	taxusin	-1.15	-0.43
	7β-hydroxytaxusin	2.18	0.1
	bis(β-D-glucosyl) crocetin		10.13
	β-D-gentiobiosyl crocetin		10.13
	β-D-glucosyl crocetin	-10.13	-0.93
	15-hydroxysolavetivone	-2.35	-1.09
	3-hydroxy-15-dihydrolubimin	-1.7	-0.55
	(-)-curcuquinone	-3.78	-1.14
	crocetin	-1.92	-0.61
	picrocrocin	10.13	10.13
	geranyl 6-O-β-D-xylopyranosyl-β-D-glucopyranoside	-10.13	-2.83
	2,7-dihydroxycadalene	-2.22	0.43
	(2E,6E)-farnesal	-10.13	1.12
	gypsogenin-28-beta-D-glucoside	-10.13	0.59
	16-alpha-hydroxygypsogenate	-1.49	-0.56
	gypsogenate	-10.13	-1.14
	9'-cis-neoxanthin	1.24	0.11
	oryzalide A	-10.13	0.83
	cis-abscisic alcohol	-4.27	-0.58
	2-trans-abscisate	-3.98	0.35
	4'-hydroxyadonixanthin	-1.14	0.05
	gibberellin A19	-10.13	-10.13
	gibberellin A24	3.68	1.43
	gibberellin A9	-10.13	-10.13
	gibberellin A6	-10.13	-1.18
	gibberellin A5	3.42	1.82
	ent-kaurenal	1.87	-10.13
	(4S)-4-(5,5-dimethylcyclohex-1-en-1-yl)cyclohex-1-ene-1-carbaldehyde	-10.13	-0.46
	ajmaline	-10.13	0.78
	17-O-acetylajmaline		10.13
	16-epivellosimine	-4.98	-3.95
	17-O-acetylnorajmaline		10.13
	dehydroabietadienal	10.13	10.13
	benzoyl-β-D-glucopyranose	2.12	0.31
	7-deoxyloganetate	10.13	
	(6E)-8-hydroxygeraniol	-10.13	2.44
	desoxyhemigossypol-6-methyl ether	-1.17	-0.55
	geranylgeranyl diphosphate (GGPP)	-3.69	0.53
	kauralexin A2	5.67	6.54
	kauralexin A1	3.78	1.65
	ent-3β-hydroxy-cassa-12,15-diene-11-one	1.18	0.11
